# Gloria Werner, 1940–2021

**DOI:** 10.5195/jmla.2021.1276

**Published:** 2021-10-01

**Authors:** Alison Bunting, J. Michael Homan

**Affiliations:** 1 alisbunting@gmail.com, Biomedical Librarian Emerita, UCLA Louise Darling Biomedical Library, Sonoita, AZ; 2 homan@mayo.edu, Director of Libraries Emeritus, Emeritus Consultant Health Sciences Research, Mayo Clinic, Rochester, MN

## Abstract

Gloria Werner, successor to Louise M. Darling at the UCLA Louise M. Darling Biomedical Library, university librarian emerita, and eighteenth editor of the *Bulletin of the Medical Library Association*, died on March 5, 2021, in Los Angeles. Before assuming responsibility in 1990 for one of the largest academic research libraries in the US, she began her library career as a health sciences librarian and spent twenty years at the UCLA Biomedical Library, first as an intern in the NIH/NLM-funded Graduate Training Program in Medical Librarianship in 1962–1963, followed by successive posts in public services and administration, eventually succeeding Darling as biomedical librarian and associate university librarian from 1979 to 1983. Werner's forty-year career at UCLA, honored with the UCLA University Service Award in 2013, also included appointments as associate university librarian for Technical Services. She was president of the Association of Research Libraries in 1997, served on the boards of many organizations including the Association of Academic Health Sciences Library Directors, and consulted extensively. She retired as university librarian in 2002.

Gloria Werner, university librarian emerita and successor to Louise M. Darling at the UCLA Louise M. Darling Biomedical Library, died on March 5, 2021, in Los Angeles.

Werner was born on December 12, 1940, in Seattle, Washington. She skipped grades a couple of times in the Seattle public schools and applied to Radcliffe, Pomona College, and Oberlin College—all of which accepted her. She chose to go to Oberlin and arrived in the small college town in Ohio at the age of sixteen. While at Oberlin, she was a French major with an art history minor, but she also had a continuing interest in music, particularly classical piano. She played a piano concerto with the University of Washington Symphony orchestra when she was only fourteen, and Oberlin's well-known music conservatory allowed her to continue her piano studies. It appears that the small liberal arts college suited her as she graduated with a BA in French in three years in 1961.

While at Oberlin, Gloria worked as an assistant at the Oberlin Art Library. Following graduation, she returned to Seattle and obtained her master's in librarianship from the University of Washington in 1962. Because of her interest in libraries, she had always intended to get a library degree. Though art history was perhaps her greatest love, it would have required at least a master's or PhD and many more years of education to become an art curator or museum director, which was something she was uninterested in pursuing at the time. In 1962, she was honored with the University of Washington School of Librarianship Award for Most Outstanding Student [1].

Before assuming responsibility for one of the largest academic research libraries in the US, Gloria began her career at the UCLA Biomedical Library. She was fond of saying that despite not having attended UCLA, she was born and raised professionally there [2]. Before library school graduation, she was offered a job at Seattle Public Library, which had the largest art history collection in the area and where she had completed an internship. Even though she had no science in her academic background and had already been offered a job at Seattle Public Library, University of Washington Library School Dean Dorothy Bevis was instrumental in convincing her to apply for an internship at the UCLA Biomedical Library. After being accepted and completing the NIH/NLM-funded Graduate Training Program in Medical Librarianship Internship in 1963, she was hired as a reference librarian by Director Louise M. Darling. Gloria also celebrated a momentous event in 1963 when she married Newton Davis Werner, a Los Angeles native who had recently completed his PhD in chemistry.

From 1963 to 1979, she assumed increasingly responsible positions in the UCLA Biomedical Library including head of reference and assistant/associate biomedical librarian for public services (Figure 1). She took a year off in 1967–1968 to work in London as librarian of the Wellcome Historical Medical Library, while her husband was completing a Fulbright Fellowship. In 1979, she succeeded Louise Darling as director of the Biomedical Library (later named the Louise M. Darling Biomedical Library by action of the UC Board of Regents), and as director the Pacific Southwest Regional Medical Library Service and Cancer Information Center. As director, Gloria added computer-assisted instruction and audiovisual services, implemented the transition from bibliographic searching by librarians to end user searching, and oversaw the physical expansion of the library. She was also designated an assistant dean of the UCLA Medical School.

**Figure 1:**
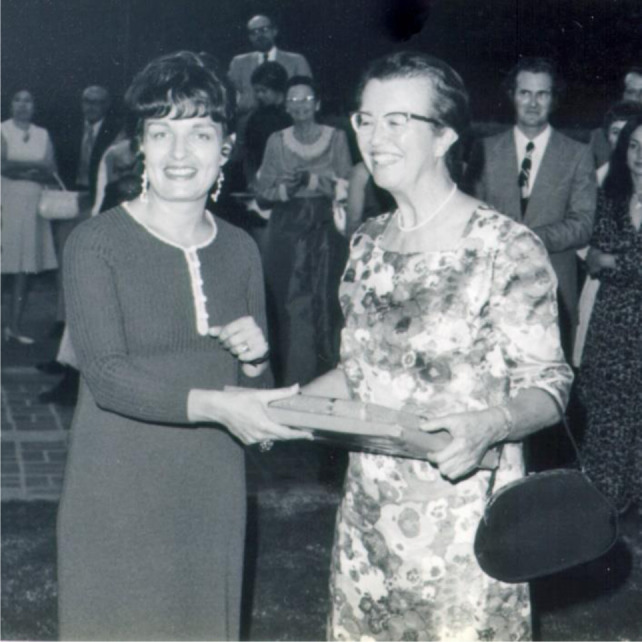
Gloria Werner (left) with Louise Darling (right), 1972

In 1983, Gloria was persuaded to take on the position of associate university librarian for technical services for the UCLA Library system. In this role, she oversaw the development of the UCLA Library's online information system, ORION, based in part on the continuation of automation efforts initiated by the Biomedical Library. She served in that capacity until 1990 when she was appointed university librarian. Her accomplishments in this position included renovating the historic Powell Library built originally as the main university library, establishing the College Library Instructional Computing Commons, managing the transition from print to electronic resources in many disciplines, reducing multiple campus library locations, and managing successive University of California budgetary shortfall issues. She also became active during this time in the Association of Research Libraries (ARL), serving as ARL President (1996–1997), as a member of the Research Collections Committee, and as a participant in ARL's Scholarly Publishing and Academic Resources Coalition (SPARC) program.

Werner was associated for ten years with publication of the Medical Library Association's journal, then titled *Bulletin of the Medical Library Association* (*BMLA*). In 1973, Robert F. Lewis, biomedical librarian at UC San Diego, was appointed to the first of two three-year terms as editor. He chose Gloria to lead the editorial committee of the journal and then, a year later, to serve as associate editor during his two terms as editor. During their tenure, the publication type called “brief communications” became part of the journal, and the editorial committee and peer review process were strengthened under Gloria's guidance. When Lewis stepped down in 1979, Werner, who was the choice of the editorial selection committee, became the eighteenth editor of *BMLA*. The editorial selection committee recommended her reappointment in 1983, but she had to decline due to her new position in the UCLA library system [3]. Werner's successor as editor praised her for “her encouragement of authors” and for “developing a peer review system that is among the best in scientific publishing” [4].

Though she was born and raised in the Pacific Northwest and arrived serendipitously at UCLA, Gloria stayed the course and contributed significantly to the development of the UCLA library system over her forty-year career. In 2013, she was honored with the UCLA University Service Award. The arc of her career spanned from MEDLARS and other batch process retrieval systems to online catalogs and digital libraries. She served on the boards of many organizations including the Association of Academic Health Sciences Library Directors and consulted extensively. She was tempted only once to return to Seattle when the University of Washington offered her the university librarian position.

When Gloria retired as UCLA university librarian in 2002, she continued to treasure her ties to UCLA as well as her love of music, art, and travel. She and her husband Newton were avid art collectors and donated generously to the Grunwald Center for the Graphic Arts in the Hammer Museum. Gloria served on the Docent Council of the Los Angeles County Museum of Art and was active in many other organizations. Music continued to be an integral part of her life as a season ticket holder of the Los Angeles Opera, Los Angeles Philharmonic, and the Ojai Music Festival. Gloria is survived by her son, Adam, daughter-in-law, Tammy, and grandson, Noah.
